# Activation of Ras-ERK Signaling and GSK-3 by Amyloid Precursor Protein and Amyloid Beta Facilitates Neurodegeneration in Alzheimer’s Disease

**DOI:** 10.1523/ENEURO.0149-16.2017

**Published:** 2017-03-27

**Authors:** Lisa Kirouac, Alexander J. Rajic, David H. Cribbs, Jaya Padmanabhan

**Affiliations:** 1 Department of Molecular Medicine, USF Health Byrd Alzheimer’s Institute, Morsani College of Medicine, University of South Florida, Tampa, FL 33613; 2 Institute for Memory Impairment and Neurological Disorders, Department of Neurology, University of California, Irvine, Irvine, CA 92697-4540

**Keywords:** A-beta, Alzheimer’s disease, amyloid precursor protein, GSK3, protein phosphorylation, Ras-MAPK signaling

## Abstract

It is widely accepted that amyloid β (Aβ) generated from amyloid precursor protein (APP) oligomerizes and fibrillizes to form neuritic plaques in Alzheimer’s disease (AD), yet little is known about the contribution of APP to intracellular signaling events preceding AD pathogenesis. The data presented here demonstrate that APP expression and neuronal exposure to oligomeric Aβ42 enhance Ras/ERK signaling cascade and glycogen synthase kinase 3 (GSK-3) activation. We find that RNA interference (RNAi)-directed knockdown of APP in B103 rat neuroblastoma cells expressing APP inhibits Ras-ERK signaling and GSK-3 activation, indicating that APP acts upstream of these signal transduction events. Both ERK and GSK-3 are known to induce hyperphosphorylation of tau and APP at Thr668, and our findings suggest that aberrant signaling by APP facilitates these events. Supporting this notion, analysis of human AD brain samples showed increased expression of Ras, activation of GSK-3, and phosphorylation of APP and tau, which correlated with Aβ levels in the AD brains. Furthermore, treatment of primary rat neurons with Aβ recapitulated these events and showed enhanced Ras-ERK signaling, GSK-3 activation, upregulation of cyclin D1, and phosphorylation of APP and tau. The finding that Aβ induces Thr668 phosphorylation on APP, which enhances APP proteolysis and Aβ generation, denotes a vicious feedforward mechanism by which APP and Aβ promote tau hyperphosphorylation and neurodegeneration in AD. Based on these results, we hypothesize that aberrant proliferative signaling by APP plays a fundamental role in AD neurodegeneration and that inhibition of this would impede cell cycle deregulation and neurodegeneration observed in AD.

## Significance Statement

Although amyloid β (Aβ) is fundamental to Alzheimer’s disease (AD) pathology development, Aβ-lowering drugs failed in clinical trials, suggesting that additional amyloid precursor protein (APP)-dependent mechanisms might play an important role in AD pathogenesis. Here we show that APP plays a significant role in the activation of Aβ-dependent Ras-MAPK signaling. Aβ fails to bring about Ras-ERK activation when APP is absent from the cells. Furthermore, we show that Ras-ERK signaling induces APP and tau hyperphosphorylation, which are enhanced in AD brains, and inhibition of Ras-MAPK activation prevents hyperphosphorylation of tau and APP as well as neuronal cell cycle entry. These results therefore demonstrate that APP is fundamental to the cell cycle deregulation and neuropathology development observed in AD.

## Introduction

Alzheimer’s disease (AD) is a progressive neurodegenerative disease that affects >5 million Americans, and its impact on the nation is reflected by its rise in incidence among the elderly and its economic burden. Whereas extracellular neuritic plaques and intracellular neurofibrillary tangles constitute the major pathologies in AD (Glenner and Wong, 1984; Masters et al. 1985; Braak and Braak, 1991, 1995; Gold et al. 2000), the molecular mechanisms that induce these pathogenic transformations are unclear. Autosomal dominant inheritance of AD is linked to mutations in genes encoding APP and presenilins 1 and 2 (Goate et al. 1991; Levy-Lahad et al. 1995; Rogaev et al. 1995; Sherrington et al. 1995) and result in increased Aβ generation, indicating its central pathogenic role in AD. Sequential cleavage of amyloid precursor protein (APP) by β- and γ-secretases yields amyloid β (Aβ) peptide (Suh and Checler, 2002), and its oligomeric species is most toxic to neurons (Lambert et al. 1998; Lesné et al. 2006; Shankar et al. 2008). The contribution of Aβ to AD pathology is understood, yet little is known about the physiologic function of the APP holoprotein or its role in AD pathogenesis.

APP phosphorylation at threonine 668 (Thr668) in the cytoplasmic domain has been shown to enhance proteolysis by β-secretase (Lee et al. 2003; Chang et al. 2006). Kinases such as GSK-3, JNK, extracellular signal–regulated kinase (ERK), cdk5, cdk4, and cdc2 phosphorylate this site on APP (Suzuki et al. 1994; Aplin et al. 1996; Iijima et al. 2000; Standen et al. 2001; Muresan and Muresan, 2005; Judge et al. 2011), and studies in AD brains have shown that compromised neurons exhibit activation of these kinases as well as aberrant expression of cyclins B, D, and E (Nagy et al. 1997; Vincent et al. 1997; Busser et al. 1998; Raina et al. 1999). Brains from AD patients also show increased levels of Thr668 phosphorylation on APP (Lee et al. 2003), but whether this phosphorylation is due to aberrant cell cycle activation is unclear. We previously demonstrated that transgenic mice expressing APP alone or APP and PS1 show ectopic expression of P-cdc2, cyclin D, and cyclin E together with enhanced phosphorylation of APP at Thr668 (Judge et al. 2011). Using APP-expressing human neuroglioma cells, we showed that this phosphorylation occurs in a mitosis-specific manner, predominantly at the G2/M phase of the cell cycle, and leads to APP centrosomal association, implying a role for APP in cell cycle regulation (Judge et al. 2011). Utilizing stable isotope labeling by amino acids in cell culture (SILAC), we showed that APP induces expression of proteins involved in cellular assembly, organization and morphology, and cell cycle (Chaput et al. 2012). We reported that cells expressing APP show enhanced Ras expression and activation of ERK1/2 (Chaput et al. 2012). These findings prompted us to hypothesize that APP or a metabolite of APP promotes Ras/mitogen-activated protein kinase (MAPK) signaling and enhanced proliferation in cells.

Primary cortical rat neurons treated with Aβ42 have shown aberrant expression of cell cycle markers, DNA replication, and mitotic catastrophe (Copani et al. 1999; Giovanni et al. 1999). Based on our findings that APP induces Ras-MAPK signaling and that mitosis-specific phosphorylation of APP at Thr668 leads to centrosome-association of APP, we hypothesize that APP or its metabolites play a fundamental role in promotion of cell cycle deregulation observed in AD. Using APP-specific small interfering RNAs (siRNAs), here we present evidence for APP-mediated activation of Ras-MAPK signaling cascade and GSK-3 in B103 neuroblastoma cells expressing APP, which are recapitulated upon treatment of neurons with Aβ42. In addition to enhanced Ras-ERK signaling and GSK-3 activation, neurons showed enhanced phosphorylation of APP and tau upon Aβ treatment, indicative of a positive feedback mechanism by which Aβ induces APP-dependent neurodegeneration. Our studies in human AD brain samples enabled us to validate these findings, demonstrating the importance of interfering with aberrant APP or Aβ-dependent signaling for prevention of cell cycle deregulation and neurodegeneration in AD.

## Materials and Methods

### Reagents and antibodies

Tissue culture reagents were purchased from Invitrogen; electrophoresis supplies were from Bio-Rad Laboratories; SuperSignal West Pico Chemiluminescent Substrate and Hoechst 33342 were purchased from Thermo Fisher Scientific; APP siRNA, control siRNA-A, and siRNA transfection reagent were purchased from Santa Cruz Biotechnology; poly-l-lysine was purchased from Sigma-Aldrich; TUNEL assay kit (*in situ* cell death detection kit, fluorescein) was purchased from Roche; and recombinant Aβ(1–42) peptide was purchased from American Peptide Company. The company, catalog number, and the Research Resource Identifiers (RRIDs) for the antibodies used in this study are as follows: Alexa Fluor 488–conjugated goat anti-mouse IgG (Thermo Fisher Scientific, A-11029, RRID:AB_2534088), Alexa Fluor 594–conjugated goat anti-rabbit IgG (Thermo Fisher Scientific, A-11012, RRID:AB_2534079), horseradish peroxidase (HRP)-conjugated goat anti-mouse IgG (Southern Biotech, 1030-05, RRID:AB_2619742), HRP-conjugated goat anti-rabbit IgG (Southern Biotech, 4010-05, RRID:AB_2632593), anti–α-tubulin (Sigma-Aldrich, T9026, RRID:AB_477593), anti–β-actin (Sigma-Aldrich, A5316, RRID:AB_476743), anti-GAPDH (Sigma-Aldrich, G8795, RRID:AB_1078991), anti–β-amyloid, 1–16 (6E10; BioLegend, 803003, RRID:AB_2564652), anti-Tau1 (Millipore, MAB3420, RRID:AB_94855), anti–Cyclin D1 (Santa Cruz Biotechnology, sc-8396, RRID:AB_627344), anti-SOS2 (Santa Cruz Biotechnology, sc-258, RRID:AB_2192448), anti–phospho-APP (Thr668; Cell Signaling Technology, 3823S, RRID:AB_2056410), anti–phospho-p44/p42 MAPK (Thr202/Tyr204; P-ERK1/2; Cell Signaling Technology, 9101, 9101S, 9101L, RRID:AB_331646), anti-p44/42 MAPK (ERK1/2; Cell Signaling Technology, 9102, 9102L, 9102S, RRID:AB_330744), anti–phospho-GSK-3α/β (Ser 21/9; Cell Signaling Technology, 9331, 9331L, 9331S, RRID:AB_329830), anti–GSK-3α/β (Cell Signaling Technology, 5676, 5676P, 5676S, RRID:AB_10547140), anti–histone H3 (Cell Signaling Technology, 9717, RRID:AB_331222), anti-Ras (Abcam, ab52939, RRID:AB_2121042), anti-Grb2 (Cell Signaling Technology, 3972S, RRID:AB_10693935), anti-MAP2 (Abcam, ab24645, RRID:AB_448210), and anti–phospho-tau (Thr231; MBL International, AT-5019, RRID:AB_843632). Anti–PHF-1 (phospho-tau Ser 396/Ser404) antibody was provided by Dr. Peter Davies (Albert Einstein College of Medicine, Manhasset, NY).

### Cell culture

APP-null B103 (RRID:CVCL_D538) and APP695 isoform–expressing B103 (B103-695) rat neuroblastoma cells were obtained from Dr. David Schubert (Salk Institute, La Jolla, CA). These cells were cultured in DMEM/Ham’s F-12 (advanced DMEM/F-12) supplemented with 10% fetal bovine serum (FBS) and 1% penicillin/streptomycin at 37˚C and 5% CO_2_ (Jin et al. 1994).

### Ras-GTP pull-down assay

The Ras-GTP pull-down assay was performed using the Ras Activation Assay Biochem Kit from Cytoskeleton, according to the manufacturer’s protocol. Briefly, B103 and B103-695 cells were grown to 80% confluence (∼3 × 10^6^ cells) in 100-mm tissue culture dishes and lysed in 400 μl of ice-cold lysis buffer supplemented with protease and phosphatase inhibitors. Lysate was cleared by centrifugation, and 300 μg of protein from each sample was collected. As a positive control, extracts were loaded with GTPγS (a nonhydrolysable GTP analog) or, as a negative control, extracts were loaded with GDP. Lysates were incubated by end-over-end rotation with 100 μg Raf-RBD conjugated beads for 1 h. Supernatant was then carefully removed and the beads were washed and boiled in 2× Laemmli sample buffer, following which Western blot analysis was performed using the pan-Ras antibody provided with the kit.

### Primary neuron culture

Timed pregnant Sprague-Dawley rats were obtained from Harlan, and E18 embryos were collected after euthanization by pentobarbital injection. Fetal brains were collected and placed in isotonic solution, meninges removed, and cortices excised. Single-cell suspension was prepared by triturating cortices with fire-polished glass pipettes in 2 ml of isotonic buffer. The neuronal suspension was then spun down at 1500 RPM for 5 min at 4°C and isotonic buffer was aspirated. The neuronal pellet was then resuspended in neurobasal medium supplemented with 2× B27, 1% penicillin/streptomycin, and 2 mm glutamine and seeded onto cell culture dishes coated with 200 μg/mL poly-l-lysine (PLL). Eight-chamber slides were plated with ∼5 × 10^4^ neurons per well, six-well plates were plated with ∼1 × 10^6^ neurons per well, and 100-mm dishes were plated with ∼6 × 10^6^ neurons. Neurons were replenished with 50% fresh medium every third day and grown for at least 5 d before treatment.

### Oligomeric Aβ preparation

We followed the protocol by Stine et al. (2011) for oligomeric Aβ preparation. We dissolved 1 mg of monomeric Aβ42 in 1 ml trifluoroacetic acid (TFA) and divided it into 100-μl aliquots. Aliquots containing ∼100 μg of Aβ were then lyophilized. The lyophilized Aβ was solubilized in sterile DMSO to obtain a concentration of 5 mm. This solution was then diluted to 100 μm in F12 medium and left at 4°C overnight to produce oligomeric Aβ42 peptide.

### Human brain samples

The brain tissues used for this project was provided by the University of California, Irvine Alzheimer’s Disease Research Center (UCI-ADRC) and the Institute of Memory Impairments and Neurologic Disorders. Brain samples were categorized based on clinical MMSE score and postmortem Braak stage. Additional information on this brain material is detailed in Table 1. Brain samples from four males and 10 females were categorized as non-AD (NAD), mild cognitive impaired (MCI), and late AD (LAD). Brain tissue was homogenized with 100 mm Tris-HCl (pH 7.6) containing 4% SDS, 100 mm dithiothreitol (DTT), and Halt protease inhibitor cocktail (Pierce). Homogenates were briefly sonicated and centrifuged for 15 min at 14,000 RPM at 4°C. The soluble supernatant fraction was collected from the insoluble pellet and used for Western blot analysis.

**Table 1.** Demographic and cognitive state of human brain tissue samples used in this study (data shown in Fig. 3)

Case no.	Age, years	Sex	Braak stage	PMI	MMSE	Diagnosis
11	86	M	2	4.42	27	NAD
34	91	F	3	3.33	30	NAD
29	83	F	4	5.25	30	NAD
41	91	F	4	4.82	29	NAD
24	86	F	3	2.92	22	MCI
17	86	F	3	6.17	30	MCI
9	87	M	5	6.17	24	MCI
35	94	M	1	3.87	27	MCI
45	95	F	5	5.30	24	MCI
12	82	F	6	5.92	17	LAD
39	90	M	6	4.17	14	LAD
37	88	F	5	4.50	10	LAD
10	82	F	6	4.58	-5	LAD
40	96	F	6	4.50	20	LAD

MMSE, Mini Mental State Examination; PMI, postmortem interval; NAD, non-AD; MCI, mild cognitive impaired; LAD, late AD.

### Aβ treatment and APP knockdown

For neuronal Aβ treatment studies, primary rat cortical neurons were treated with 2.5 or 5 μm oligomeric Aβ42 for 24 h. DMSO treatment served as a vehicle control. For low-concentration Aβ treatments, neurons were incubated with 10–100 nm Aβ for 24–144 h, and lysates were prepared and analyzed by Western blot. For APP knockdown studies, B103-695 cells were grown to 60% confluence in six-well plates with penicillin/streptomycin-free advanced DMEM/F-12. Cells were then transfected with 30, 40, 60, 90, and 120 nm of APP siRNA or 120 nm scrambled siRNA-A following the manufacturer’s protocol (Santa Cruz Biotechnology). Seven hours after transfection, medium was replenished with an equal volume of advanced DMEM/F-12 containing 2× serum and cultured for 48 h before harvesting.

### Inhibitor treatment

B103-695 cells were treated with the MEK inhibitor U0126 (10 μm), the farnesyl transferase inhibitor tipifarnib (5 μm, for Ras inhibition), the transcriptional inhibitor aphidicolin (5 μg/ml), or the cell cycle inhibitor roscovitine (20 μm) for 24 h. Cell lysates were prepared and examined for changes in P-ERK, Ras, and cyclin D1 by Western blot analysis. Similarly, primary rat cortical neurons were treated with or without 10 μm U0126, 5 μm tipifarnib, 5 μg/ml aphidicolin, or 20 μm roscovitine in the presence or absence of 2.5 μm Aβ for 24 h and analyzed by immunostaining, as described below, for cellular levels and distribution of Ras, P-ERK, cyclin D1, and P-APP using the corresponding antibodies. MAP2 antibody was used as neuronal marker.

### Immunostaining

Primary neurons were fixed with 4% paraformaldehyde for 10 min at room temperature, rinsed three times with 1× PBS, and incubated for 1 h in 1× TBS with 10% normal goat serum (NGS) and 0.2% Triton X-100 blocking buffer. Neurons were then incubated overnight at 4°C with P-Thr668 APP (1:1000), cyclin D1 (1:250), P-ERK (1:500), or Ras (1:500) rabbit primary antibody, and MAP2 (1:1000) mouse primary antibody, diluted in 1× TBS with 1% bovine serum albumin (BSA) containing 0.1% Triton X-100. After incubation, cells were washed with 1× PBS four times, 5 min each. Cells were then incubated for 2 h at room temperature with goat anti-mouse IgG Alexa Fluor 488 (1:1000) and goat anti-rabbit IgG Alexa Fluor 594 (1:4000) diluted in blocking buffer. After two washes, cells were incubated with 1 μg/mL Hoechst 33342 DNA dye diluted in 1× PBS for 5 min. After several washes, the slides were coverslipped with Fluoro-Gel mounting media and analyzed under a Zeiss Fluorescence Axio Imager using AxioVision Rel 4.8 software.

### Western blot analysis

Cells were harvested in their media and pelleted at 1500 RPM for 5 min at 4°C. Medium was discarded with the exception of neuronal media, which was diluted with a final concentration of 1× Laemmli’s sample buffer (to validate Aβ42 levels and oligomerization). The pellet was resuspended in 1× PBS and washed three times with subsequent centrifugation. Cell pellets were then lysed with 1× RIPA lysis buffer containing protease and phosphatase inhibitors (1 mm PMSF, 1 mm NaF, 1 mm sodium orthovanadate) and complete miniprotease inhibitor (Roche Diagnostics). Cell lysates were placed on ice for 30 min, further lysed by sonication, and then pelleted by centrifugation at 14,000 RPM for 15 min. Pellet was separated from supernatant and protein measured using Pierce 660-nm Protein Assay Reagent (Thermo Fisher Scientific). Equal amounts of protein were diluted and resuspended in a final concentration of 1× Laemmli’s sample buffer. Samples were boiled for 5 min, separated on 12% Tris-glycine gels, and electrotransferred to nitrocellulose membranes (GE Healthcare). Membranes were incubated in 5% nonfat dairy milk in 1× TBS for 1 h at room temperature to inhibit nonspecific binding. Blots were then incubated with primary antibody overnight at 4°C, followed by four 5-min washes in 1× PBS with 0.05% Tween 20 (PBST). Blots were then incubated with appropriate secondary antibody conjugated to HRP. Immunoreactivity was detected using SuperSignal West Pico Chemiluminescent Substrate and captured on autoradiography film (MidSci) or imaged using a GE Healthcare 600 Chemiluminescence Imager. Results were quantified using ImageJ.

### Extraction of nuclear and cytoplasmic proteins from neurons

For separation of nuclear and cytoplasmic protein from neurons, we used the slightly modified protocol by Dignam et al. (1983). Briefly, cells were washed with cold PBS, pelleted by centrifugation at 1000 rpm for 5 min, pellet was resuspended in 5× volume of ice-cold buffer A containing 10 mm Hepes, pH 7.9, 1.5 mm MgCl_2_, 10 mm KCl, 1 mm DTT, and 0.1% NP-40, incubated on ice for 5 min, and passed through a 27-gauge needle 30 times. Lysate (5 μl) was mixed with Trypan blue and analyzed under the bright microscope to confirm that all cells were broken and the nuclei were separated. The lysate were spun at 3000 rpm for 5 min, and the supernatant was collected as cytoplasmic extract. The nuclear pellet was washed three times by resuspending in buffer A without NP-40 and centrifuging at 3000 rpm. The nuclei are resuspended in Laemmli sample buffer and boiled for 5 min to solubilize the nuclear proteins, which was used as nuclear extract. Both cytoplasmic and nuclear extracts were analyzed by Western blotting for alterations in Ras and P-ERK in neurons treated with or without Aβ in the presence or absence of U0126 and Tipifarnib.

### TUNEL assay for apoptosis detection in neurons

To determine the extent of apoptosis under treatment with Aβ alone or in the presence or absence of U0126 and Tipifarnib, we conducted TUNEL assay using the TUNEL staining kit from Roche according to the manufacturer’s instruction. Briefly, cells were treated with Aβ with or without the inhibitors for 24 h, then fixed with 4% paraformaldehyde in PBS for 10 min at room temperature and washed with PBS. Cells were incubated with freshly prepared permeabilization buffer (0.1% Triton X-100 in 0.1% sodium citrate) for 2 min on ice. The TUNEL reaction mixture was then added to the cells and incubated at 37°C for 1 h. For negative control, cells were incubated with only label solution that was not mixed with the enzyme. After incubation, cells were washed and counterstained with Hoechst by incubating for 5 min at room temperature with 1 µg/ml Hoechst 33342 (Sigma-Aldrich) diluted in PBS. The slides were washed and mounted using fluoromount and analyzed for TUNEL positivity under the fluorescent microscope.

### Statistics

Statistical analysis was performed using Student’s *t* test for two-group comparisons or, for multiple comparisons, ANOVA was performed. All experiments were independently performed ≥3 times.

## Results

### B103-695 cells show increased levels of active Ras

**Figure 1.**
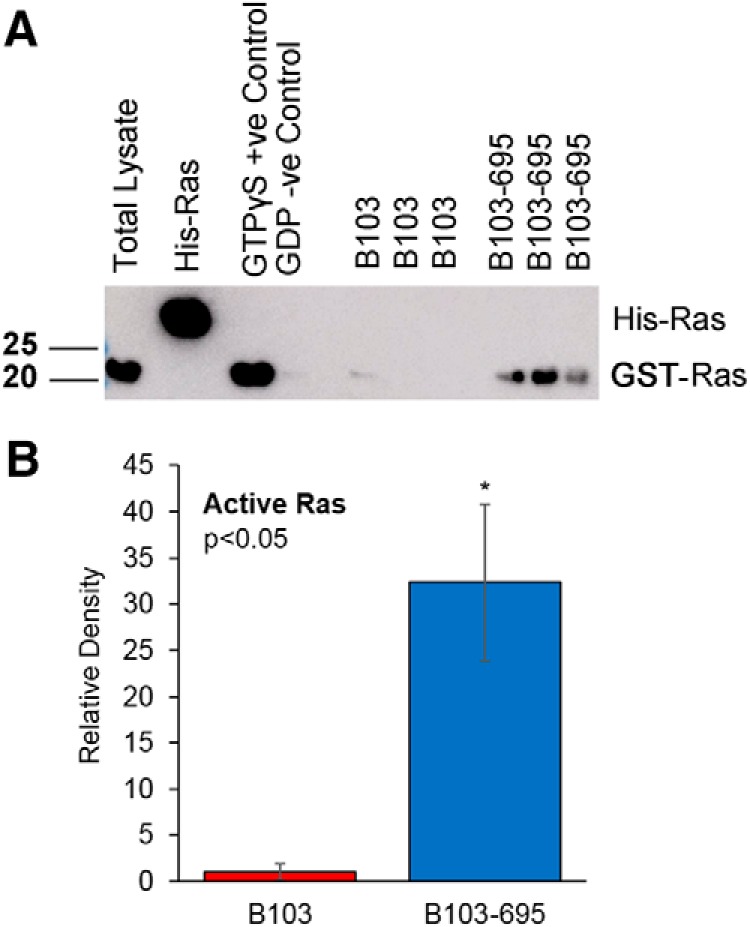
Analysis of APP-null B103 and APP-expressing B103-695 cells show increased levels of active Ras in B103-695 cells. B103 and B103-695 cells were analyzed for GTP-bound Ras by Ras activation assay and analyzed by Western blot. Extract (20 μg) of cell lysate was loaded in Lane 1; 20 ng of His-Ras control protein was loaded in Lane 2; a positive control of 300 μg lysate loaded with nonhydrolysable GTP analog (GTPγS) is in Lane 3; a negative control of 300 μg lysate loaded with GDP is in Lane 4; Lanes 5–7 are B103 extracts (300 μg) incubated with 30 μl Raf-RBD beads; Lanes 8–10 are B103-695 extracts (300 μg) incubated with 30 μl Raf-RBD beads. ***A***, The membrane was probed with antibody to Ras, provided with the kit, to confirm pull down of active Ras. ***B***, The bar graph represents the quantitative analysis of levels of active Ras. Statistical analysis was performed using Student’s *t* test. The data represent the mean ± SEM from three independent experiments (*n* = 3); *p < 0.05.

Previously, using SILAC-based proteomic analysis, we have shown that B103 cells expressing APP-695 differentially express a number of proteins compared with their B103 APP-null counterparts. We found that B103-695 cells express significantly higher levels of Ras and show activation of ERK1/2 (Chaput et al. 2012), indicative of activation of Ras-MAPK signaling axis under APP-expressing conditions. To confirm that APP expression correlates with not only increased expression but also activation of Ras, we conducted a Ras-GTP pull-down assay. Ras is known to interact with its guanine nucleotide exchange factor (GEF), where it facilitates Ras activation by catalyzing the conversion of inactive Ras-GDP to active Ras-GTP (Prior and Hancock, 2012). The Ras-GTP pull-down assay uses the Ras effector protein, Raf kinase, to recognize Ras in its active, GTP-bound state. The Ras-binding domain (RBD) of Raf is conjugated to beads to specifically bind and pull down the active form of Ras. Western blot analysis was performed on the pulled-down proteins using a pan-Ras antibody provided with the kit to detect Ras-GTP. As expected, B103 cells showed very little to no Ras-GTP, whereas B103-695 cells exhibited significantly more active Ras (Fig. 1*A*, *B*). These findings confirmed that APP expression not only enhances expression but also activation of Ras. These findings agreed with our published results (Chaput et al. 2012), where we showed that APP-expressing B103 cells exhibit increased expression of Ras and phosphorylation of ERK, indicative of activation of the Ras-ERK signaling axis in the cells.

### APP expression enhances proliferative signaling and GSK-3 activation

**Figure 2.**
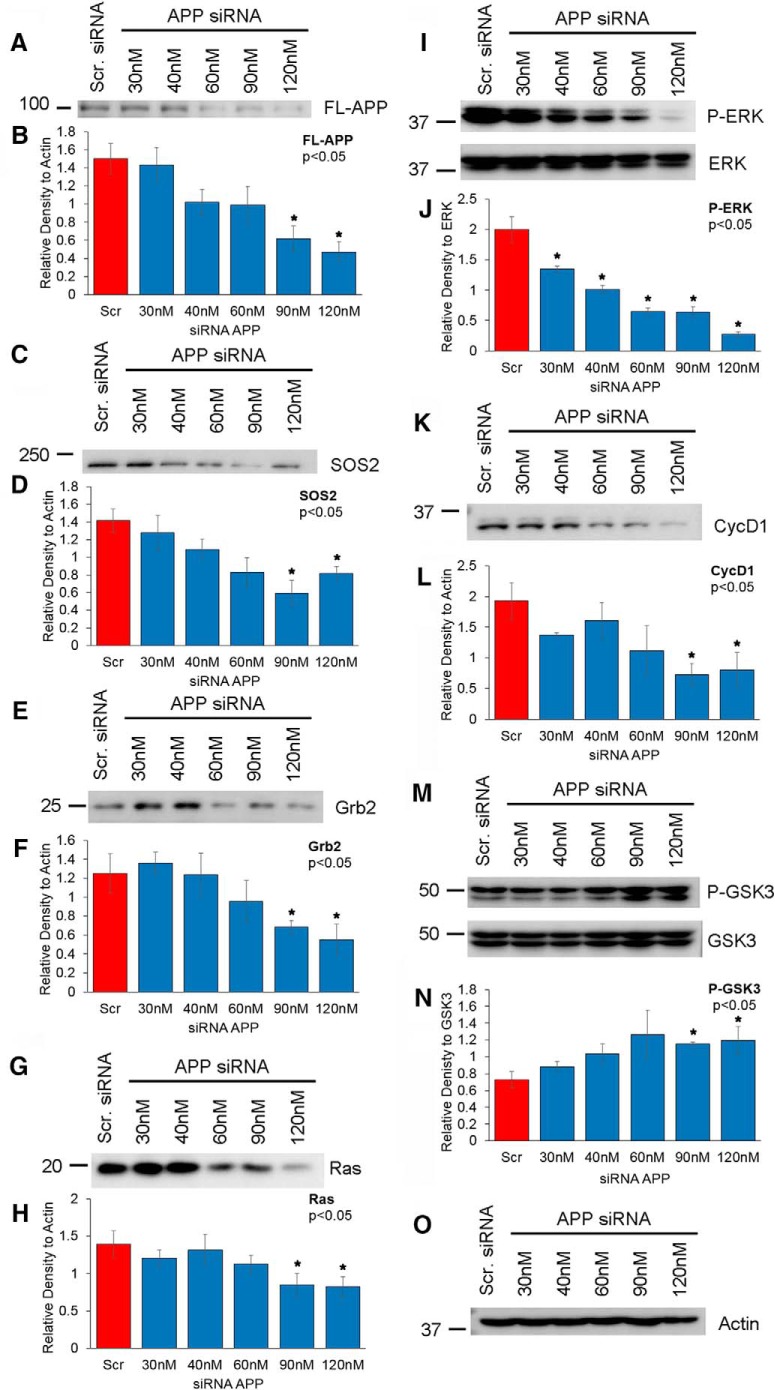
Knockdown of APP in B103-695 cells results in decreased Ras-MAPK signaling and GSK-3 activity. B103-695 cells were transfected with increasing concentrations of APP siRNA ranging from 30 to 120 nm or with 120 nm scrambled siRNA-A. At 48 h after transfection, cell extracts were prepared and analyzed by Western blot. ***A***, The membrane was initially probed with antibody to 6E10 to visualize knockdown efficiency. The membrane was then sequentially probed with SOS2 (***C***), Grb2 (***E***), Ras (***G***), P-ERK and total ERK (***I***), CycD1 (***K***), P-GSK3αβ and total GSK3αβ (***M***) antibodies. Finally, the membrane was probed with antibody to Actin (***O***), which served as loading control. The histograms represent the quantitative analysis of protein levels normalized to Actin; full-length APP (***B***), SOS2 (***D***), Grb2 (***F***), Ras (***H***), and CycD1 (***L***), respectively. Histograms representing the quantitative analysis of P-ERK and P-GSK3 normalized to corresponding total ERK or GSK3 proteins are also shown (P-ERK [***J***] and P-GSK3 [***N***]). Statistical analysis was performed using Student’s *t* test. The data represent the mean ± SEM from seven independent experiments (*n* = 7); *p < 0.05.

To confirm our findings that Ras-ERK signaling occurs downstream of APP, we used an RNA interference (RNAi) approach to downregulate APP expression in B103-695 cells and examined whether APP knockdown affects Ras expression and ERK phosphorylation. B103-695 cells were transfected with incremental concentrations (30 to 120 nm) of siRNA targeting APP or with a nontargeting, scrambled control siRNA-A (120 nm). At APP siRNA concentrations of 90 nm and higher, the B103-695 cells showed significant decrease in the levels of APP compared with control siRNA transfected cells (Fig. 2*A*, *B*). At siRNA concentrations that showed significant APP downregulation in B103-695 cells, a significant decrease in son of sevenless 2 (SOS2), an effector protein that enhances the GDP→GTP exchange and activation of Ras (Fig. 2*C*, *D*), Grb2 (Fig. 2*E*, *F*), Ras (Fig. 2*G*, *H*), and cyclin D1 (Fig. 2*K*, *L*) were also observed. ERK activation, as indicated by phosphorylation, showed a significant decrease even at the lowest APP siRNA concentrations tested (Fig. 2*I*, *J*). Total levels of ERK were not affected by APP knockdown (Fig. 2I, lower panel), and actin was used as a loading control (Fig. 2O). These findings confirm that APP is indeed an upstream modulator of the proliferation-associated Ras-MAPK signaling cascade. *In vitro* studies have identified that the Lys-Ser-Pro motifs in tau are substrates for ERK1/2 phosphorylation (Drewes et al. 1992; Goedert et al. 1992), and it is therefore possible that APP plays a role in regulation of this pathway and the subsequent pathogenic phosphorylation of tau. Another kinase involved in tau phosphorylation is GSK-3. GSK-3α and β significantly contribute to tau hyperphosphorylation at both primed and unprimed phosphorylation sites (Hanger et al. 1992; Lovestone et al. 1994; Cho and Johnson, 2003). Interestingly, APP intracellular domain (AICD) has been shown to affect GSK-3β expression and activity (Zhou et al. 2012); therefore, we next examined whether APP knockdown affects GSK-3 activity or expression. We found that phosphorylation at Ser9 and Ser21 on GSK3β and GSK3α, respectively, was significantly increased upon APP downregulation, whereas total GSK-3 levels remained unchanged (Fig. 2*M*, *N*). Because these specific phosphorylations are known to inhibit the activity of the respective GSK-3 kinases, the observed increase in phosphorylation upon APP downregulation indicates that APP or a metabolite of APP is capable of regulating GSK-3 activity.

### Brain samples from AD patients show increased Ras expression, GSK-3 activation, and APP and tau phosphorylation

**Figure 3.**
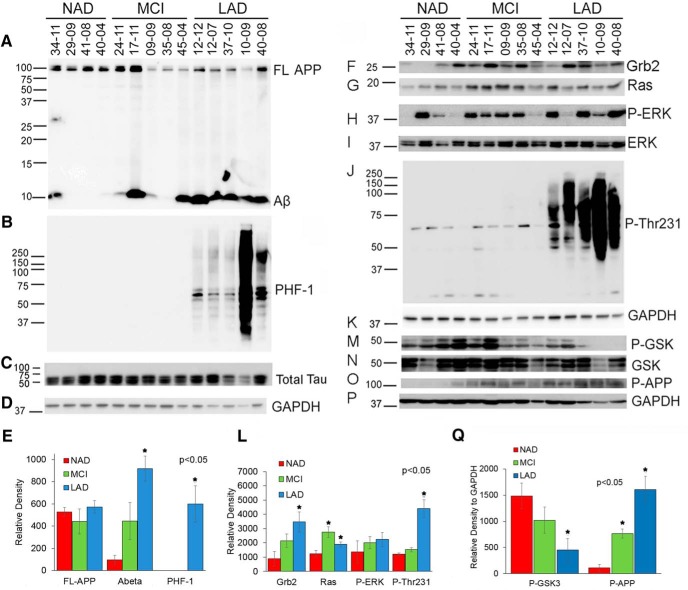
Human brain samples from MCI and LAD show increased expression of Ras, activation of GSK-3αβ, and phosphorylation of APP and tau. Brain lysates prepared from the superior frontal gyrus of MCI, LAD, and control NAD subjects were analyzed by Western blot using the indicated antibodies. To confirm disease state, membranes were probed with 6E10 antibody to detect both FL-APP and Aβ (***A***), PHF-1 antibody to detect phosphorylated tau (***B***), and Tau 1 antibody to detect total tau (***C***). ***D***, Finally, blots were probed with antibody to GAPDH, which served as a loading control. ***E***, The bar graphs represent the quantitative analysis of protein levels normalized to GAPDH. Additional sets of membranes were probed with Grb2 (***F***), Ras (***G***), P-ERK (***H***), and after stripping, nonphospho ERK antibody to detect total ERK expression (***I***) and P-Thr231 tau (***J***). ***K***, The membrane was then probed with GAPDH antibody, which served as loading control. ***L***, The bar graph represents the qualitative analysis of the normalized proteins. Analyses were also done using antibodies to P-GSK3αβ (***M***), total GSK3αβ (***N***), and P-Thr668 APP (***O***). ***P***, Membranes were reprobed with GAPDH antibody, which served as loading control. ***Q***, The bar graph represents the quantitative analysis of protein levels normalized to the corresponding total protein GSK3 or to GAPDH. Statistical analysis was performed using one-way ANOVA. The data represent the mean ± SEM; NAD, *n* = 4; MCI, *n* = 5; and LAD, *n* = 5; *p < 0.05.

Next, we sought to determine whether the APP-dependent changes in expression of proliferation-associated proteins that we observed in B103-695 cells would translate to protein expression in the human AD brains. Protein extracts from the postmortem superior frontal gyrus of control NAD, MCI, and LAD patients were examined by Western blot analysis. Disease state was confirmed by probing with 6E10 antibody, detecting full-length APP and Aβ, and PHF-1 antibody, detecting hyperphosphorylation of tau at Ser396/Ser404. Although expression levels of full-length APP showed no significant difference between disease states, Aβ generation was significantly higher in the LAD individuals compared with MCI and control (Fig. 3*A*, *E*); it is possible that the enhanced proteolysis and generation of Aβ prevents full-length APP from accumulating in the brain. Similar to Aβ, PHF-1 also showed significant increase in LAD brain, with the samples showing higher and lower molecular weight bands with PHF-1 antibody (Fig. 3*B*, *E*). Total level of tau seems to be unaltered between the NAD, MCI, and LAD brain samples (Fig. 3*C*). Blots reprobed with GAPDH antibody were used for normalization (Fig. 3*D*). Analysis of the adaptor protein Grb2 showed significant elevation in the LAD individuals (Fig. 3*F*, *L*). In the MCI and LAD individuals, Ras expression was significantly elevated compared with control samples (Fig. 3*G*, *L*). Levels of phosphorylated ERK1/2 (P-ERK) normalized to total ERK showed a trend toward increase but showed inconsistencies within disease states and did not reach significance compared with the levels in NAD samples (Fig. 3*H*, *I*, *L*). Ras expression and activation are tightly controlled, as it can induce malignant transformation in nonneuronal cells. The increased Ras expression in MCI and AD brains, together with our findings from the APP-expressing cells, suggests that aberrant Ras expression might enhance proliferative signaling. Studies from others have shown that brains from MCI patients show increased expression of Ras, and Ras expression enhances dedifferentiation of neurons (Arendt et al. 2000). A growing body of evidence indicates that the human AD brain exhibits cell cycle dysregulation and show aberrant expression of cell cycle regulatory proteins (Nagy et al. 1997; Vincent et al. 1997; Busser et al. 1998; Raina et al. 1999). Additionally, AD brains show increased APP phosphorylation at Thr668, which is known to be phosphorylated by kinases such as GSK-3, cdk5, ERK, JNK, and cdc2 (Suzuki et al. 1994; Aplin et al. 1996; Iijima et al. 2000; Standen et al. 2001; Muresan and Muresan, 2005; Judge et al. 2011). Our lab previously demonstrated that brains from transgenic mouse models of AD show altered expression of P-cdc-2, cyclin D1, and cyclin E, together with enhanced phosphorylation of APP at Thr668 (Judge et al. 2011). As shown by others (Lee et al. 2003), we also find that the brain samples from LAD patients show enhanced phosphorylation of APP at Thr668 (Fig. 3*O*, *Q*). Additionally, the inhibitory phosphorylations at Ser21 and Ser9 on GSK-3αβ are decreased in LAD samples, indicative of activation of these kinases (Fig. 3*M*, *Q*). Total GSK-3 appeared to be unaltered (Fig. 3*N*). GSK-3 is a tau kinase and its activation has been associated with phosphorylation at PHF-1 sites as well as the Thr231 priming site on tau (Goedert et al. 1994; Cho and Johnson, 2003, 2004). Analysis of the blots with P-Thr231 tau antibody showed that phosphorylation at this tau epitope was also significantly higher in the brain samples from the LAD patients compared with the control subjects (Fig. 3*J*, *L*). Increased Ras expression and activation of GSK-3 correlated with Aβ levels in the brain samples, implying that Aβ might elicit its toxic effects by modulating Ras-MAPK signaling and GSK3 activation, subsequently enhancing APP and tau phosphorylation and neuritic plaque and neurofibrillary tangle pathology development in AD. Blots were reprobed with GAPDH antibody for protein normalization (Fig. 3*P*).

### Oligomeric Aβ42 treatment induces proliferative signaling in primary neurons

**Figure 4.**
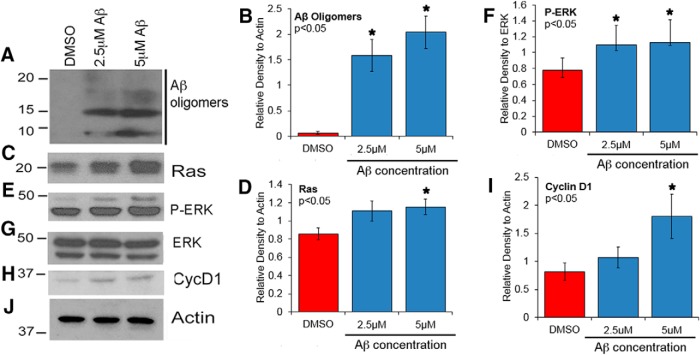
Oligomeric Aβ42 treatment induces expression of Ras and Cyclin D1 and activation of ERK in primary rat cortical neurons. Primary rat cortical neurons were cultured from E18 timed pregnant rats for at least 5 d and treated with 2.5 or 5 µm oligomeric Aβ42 for 24 h. DMSO treatment served as vehicle control. ***A***, Western blot analysis using 6E10 antibody shows presence of small Aβ oligomers in the neuronal culture supernatant. Neuronal extracts were prepared and analyzed by Western blot using Ras (***C***), P-ERK (***E***), total ERK (***G***), and cyclin D1 (***H***) antibodies. Finally, membranes were probed with Actin antibody (***J***) for protein loading on the membranes. The histograms represent the quantitative analysis of oligomeric Aβ (***B***), Ras protein expression normalized to Actin (***D***), P-ERK levels normalized to total ERK expression (***F***), and cyclin D1 expression normalized to Actin (***I***). Statistical analysis was performed using Student’s *t* test. The data represent the mean ± SEM from three independent experiments (*n* = 3); *p < 0.05.

Because Aβ levels appeared to correlate with Ras expression in AD brains, we examined whether Aβ induces Ras-ERK signaling in the neurons. Toward this we treated primary rat cortical neurons with oligomeric Aβ42 and analyzed for alterations in expression of Ras and activation of ERK. Our Aβ preparation consisted of small oligomers (Fig. 4*A*, *B*). Neurons were treated with either vehicle (0.1% DMSO) or 2.5 and 5 μm Aβ for 24 h. TUNEL analysis showed that ∼15%–20% of the neurons undergo apoptosis upon treatment with Aβ at 2.5 μm. At the end of the treatment, whole-cell lysates were prepared from control and Aβ-treated neurons and analyzed by Western blot. Results showed that Ras levels were significantly increased with 5 μm Aβ treatment (Fig. 4*C*, *D*), whereas P-ERK levels were significantly increased at both 2.5 and 5 μm concentrations (Fig. 4*E*, *F*). Total levels of ERK were unchanged (Fig. 4*G*). These data suggest that Aβ is able to induce the Ras-MAPK signaling cascade in neurons. The Ras-MAPK pathway is known to stimulate AP-1–dependent transcription of cyclin D1 (Balmanno and Cook, 1999), so next we determined whether cyclin D1 expression was also induced upon Aβ treatment. Similar to Ras and P-ERK, our results showed that at 2.5 and 5 μm Aβ concentrations, expression of cyclin D1 was increased, and at 5 μm, the increase was significant (Fig. 4*H*, *I*). The protein load on the blot was examined by reprobing with actin antibody (Fig. 4*J*).

**Figure 5.**
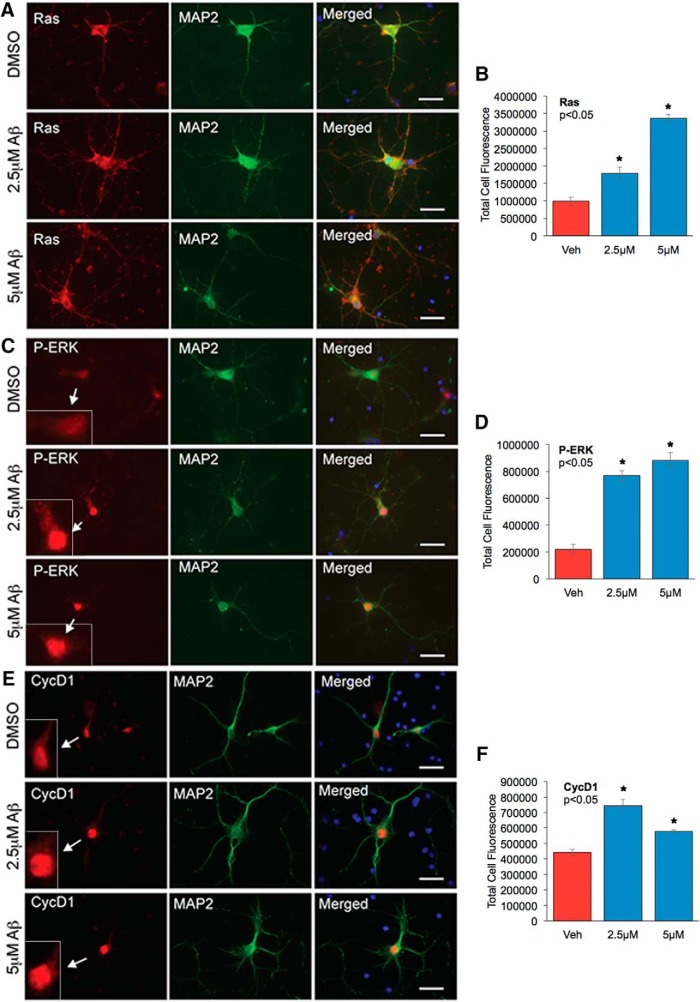
Analysis of cellular distribution of Ras and P-ERK in neurons treated with oligomeric Aβ42. Primary rat cortical neurons from E18 embryos were cultured for at least 5 d and treated with 2.5 or 5 µm oligomeric Aβ42 for 24 h. DMSO treatment served as vehicle control. Neurons were immunostained with neuron-specific MAP2 mouse monoclonal antibody and Ras (***A***), P-ERK (***C***), or Cyclin D1 (***E***) rabbit polyclonal antibodies. Arrow points to the magnified image of the cell to show nuclear staining of P-ERK or Cyclin D1. Immunostaining was visualized with Alexa Fluor 594 (red) and 488 (green) fluorophores, respectively. Hoechst staining was used to visualize nuclei (blue). The staining was analyzed with AxioVision Rel 4.8 software for Zeiss microscope. The bar graphs represent the qualitative analyses of the corrected total cell fluorescence for Ras (*n* = 30 neurons/group) (***B***), P-ERK (*n* = 30 neurons/group) (***D***), and Cyclin D1 (*n* = 30 neurons/group) (***F***). Statistical analysis was performed using ANOVA, and the data are representative of three independent experiments. Magnification 63×.

The MAPK pathway becomes activated in response to a number of stimuli that mediate a signaling cascade from the cell surface to the nucleus, and the subcellular distributions of Ras, P-ERK, and cyclin D1 play a regulatory role in their functions. To determine whether cellular distribution of Ras, P-ERK, or cyclin D1 is affected by Aβ, we treated neurons with oligomeric Aβ42 and conducted immunostaining analysis using an antibody against the neuronal marker MAP 2, together with antibodies to Ras, P-ERK, and cyclin D1. Our results showed that upon Aβ treatment, cellular levels of Ras are increased (Fig. 5*A*, *B*), similar to the results from the Western blot analysis. Analysis of P-ERK staining showed that neurons treated with 2.5 μm Aβ had increased cytosolic and nuclear staining, whereas at 5 μm Aβ, P-ERK staining was mainly observed in the nuclear compartment (Fig. 5*C*, *D*), indicative of its activation. Similarly, neurons also showed an increase in the total and nuclear levels of cyclin D1 upon treatment with Aβ (Fig. 5*E*, *F*). Nuclear cyclin D1 is associated with cell cycle activation and G1/S progression (Baldin et al. 1993). Our finding that Aβ enhances Ras-ERK signaling and increases nuclear cyclin D1 levels suggest that this would be one of the mechanisms by which Aβ enhances cell cycle deregulation, contributing to the neurodegeneration and neuronal loss observed in the AD brains.

### Oligomeric Aβ42 induces not only GSK-3 activation and Tau phosphorylation but also APP phosphorylation in neurons

**Figure 6.**
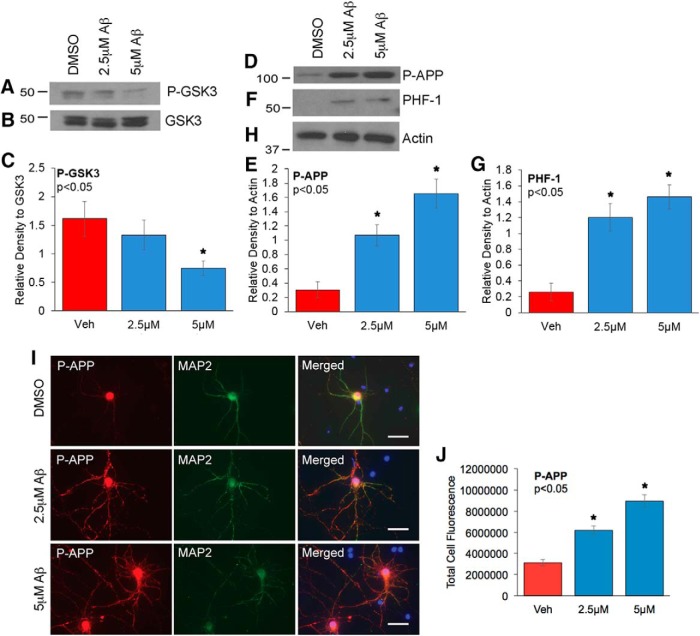
Oligomeric Aβ42 treatment induces GSK-3 activation and pathogenic phosphorylation of both APP and tau in primary rat cortical neurons. Primary rat cortical neurons were treated with 2.5 or 5 µm oligomeric Aβ42 for 24 h, and neuronal lysates were analyzed by Western blot using P-GSK3αβ (***A***) and total GSKαβ (***B***) antibodies. ***C***, The bar graph represents the quantitative analysis of P-GSK3αβ levels normalized to total GSK3αβ expression. Membranes were then probed with P-Thr668 APP (***D***) and phospho-tau PHF-1 (***F***) antibodies. Finally, the membranes were reprobed with Actin antibody (***H***), which served as loading control. The bar graphs represent the quantitative analysis of P-Thr668 APP levels normalized to Actin (***E***) and PHF-1 levels normalized to Actin (***G***). Statistical analysis was performed using Student’s *t* test, and the data represent the mean ± SEM from three independent experiments (*n* = 3); *p < 0.05. ***I***, Neurons were treated with indicated concentrations of Aβ and immunostained with neuron-specific MAP2 mouse monoclonal antibody and P-Thr668-APP rabbit polyclonal antibody. Immunostaining was visualized with Alexa Fluor 594 (red) and 488 (green) fluorophores, respectively. Hoechst staining was used to visualize nuclei (blue). The staining was analyzed with AxioVision Rel 4.8 software for Zeiss microscope. The corresponding bar graph (***J***) represents the qualitative analysis of the corrected total cell fluorescence for P-APP (*n* = 30 neurons/group). Statistical analysis was performed using ANOVA, and the data are representative of three independent experiments. Magnification 63×.

Aβ has been shown previously to induce GSK3 activation and tau hyperphosphorylation, but its role in APP phosphorylation is not established. To test this, we treated the primary rat cortical neurons with oligomeric Aβ42 at 2.5 or 5 μm, with DMSO treatment serving as control, for 24 h, and analyzed for changes in GSK3αβ and tau as well as APP. Our results showed that upon 5 μm Aβ treatment, Ser21 and Ser9 inhibitory phosphorylations on GSK-3αβ were significantly decreased, which is indicative of GSK-3 kinase activation, whereas total levels were not affected (Fig. 6*A–C*). We then examined levels of phosphorylated APP and tau, both substrates for GSK-3, and found that with 2.5 and 5 μm Aβ, there was significant increase in P-Thr668 APP and PHF-1 levels compared with vehicle-treated neurons (Fig. 6*D–G*). Blots reprobed with actin antibody were used for protein normalization (Fig. 6*H*). *In vitro* and *in vivo* studies have demonstrated that Aβ peptides can induce tau phosphorylation and subsequently lead to microtubule destabilization, impaired axonal transport, and neuronal death (Busciglio et al. 1995; Le et al. 1997; Geula et al. 1998; Takashima et al. 1998; Götz et al. 2001; Muresan and Muresan, 2009). To determine whether Aβ affects cellular distribution of phospho-APP, we performed immunostaining analysis of neurons treated for 24 h with 2.5 or 5 μm oligomeric Aβ42, using antibodies specific to the neuronal marker MAP-2 and P-Thr668 APP. Our results showed increased P-Thr668 APP staining in neurons treated with Aβ compared with DMSO-treated control neurons (Fig. 6*I*, *J*). Notably, in Aβ-treated neurons, P-Thr668 APP staining extended out into the neurites, where it appeared to accumulate in a beaded pattern (Fig. 6*I*). Neurons exposed to Aβ have been shown to exhibit neurite beading and degeneration, and it is quite possible that Aβ-mediated APP phosphorylation and accumulation in the neurites exacerbates the cytotoxic effects of Aβ on neurons.

**Figure 7.**
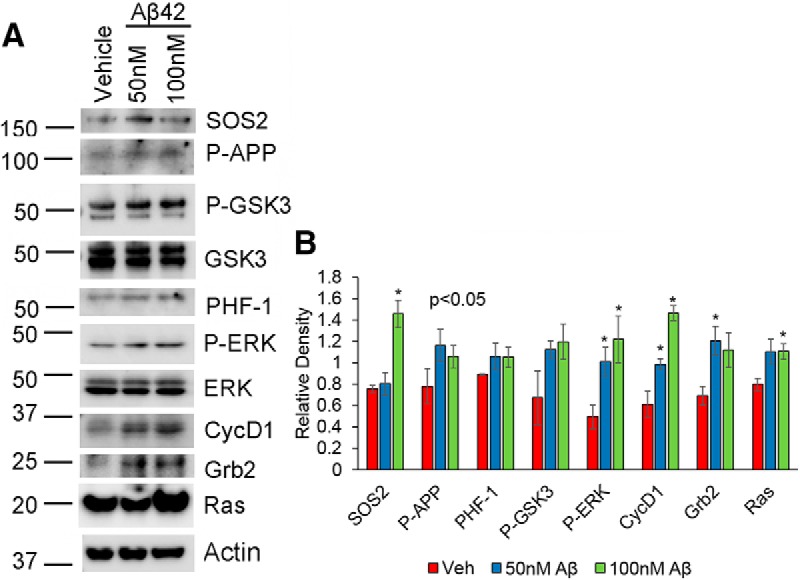
Low-concentration Aβ treatment enhances proliferative signaling in neurons. ***A***, Neurons were treated with 100 nm Aβ for 144 h, and samples were analyzed for changes in expression of the indicated proteins by Western blot. ***B***, Quantification of data from four independent experiments is shown. Statistical analysis was performed using Student’s *t* test. The data represent the mean ± SEM (*n* = 4); *p < 0.05.

To determine whether low concentrations of Aβ bring about similar changes as seen with micromolar concentrations of Aβ, we treated neurons with 10, 25, 50, and 100 nm Aβ for 24, 48, 72, 96, and 144 h and analyzed for changes in Ras, SOS2, Grb2, P-GSK3, P-ERK, cyclin D1, P-APP, and PHF-1 levels. Unlike the results from higher concentrations of Aβ, we were unable to determine any significant changes in the expression of these proteins at 24, 48, 72, or 96 h with low Aβ treatment (data not shown). We observed a significant increase in SOS2, P-ERK, cyclin D1, Ras, and Grb2 after 144 h of treatment with 100 nm Aβ (Fig. 7*A*, *B*). A concentration of 50 nm showed an increase in P-ERK, cyclin D1, and Grb2 after treatment for 144 h, whereas PHF-1 and P-APP showed a trend toward an increase. Additionally, GSK-3αβ did not show any activation at any of these time points or concentrations, implying that sustained exposure to low concentrations of Aβ may mainly enhance proliferative signaling in neurons. This agrees with other reported studies in which low concentrations of Aβ have been shown to promote neuronal cell cycle entry (López-Toledano and Shelanski, 2004; Majd et al. 2008).

### Aβ-mediated activation of the Ras-MAPK signaling axis is inhibited by the MEK inhibitor U0126 and farnesyl transferase inhibitor tipifarnib

**Figure 8.**
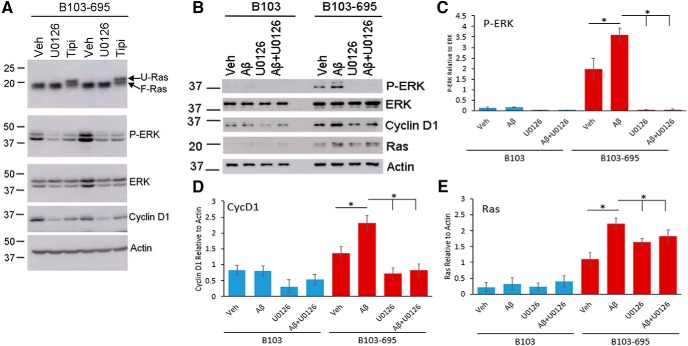
Aβ-mediated Ras-MAPK signaling and Cyclin D1 expression in B103 cells are dependent on APP expression and can be reversed with MEK or Ras inhibition. Initial studies were conducted with B103-695 cells to determine the concentration of the inhibitors required to inhibit ERK or Ras. ***A***, Treatment with 10 μm U0126 or 5 μm tipifarnib for 24 h showed unfarnesylation of Ras (U-Ras) on treatment with tipifarnib but not with U0126. Both U0126 and tipifarnib inhibited ERK activation and Cyclin D1 expression. Total ERK and actin were used for normalization of blots. Data shown is in duplicate and is representative of four independent experiments. ***B***, B103 and B103-695 cells were treated with or without 2.5 μm Aβ, in the presence or absence of MEK inhibitor U0126 for 24 h, and samples were analyzed for changes in P-ERK, ERK, Cyclin D1, and Ras by Western blot. Protein was normalized to levels of Actin. Quantification of data for P-ERK (***C***), Cyclin D1 (***D***), and Ras (***E***) are shown in respective bar graphs. Data from three independent experiments is shown. Statistical analysis was performed using ANOVA. The data represent the mean ± SEM; *p* < 0.05.

To determine whether APP-dependent expression of Ras and activation of ERK can be attenuated by the inhibitor treatment, we conducted studies on B103-695 cells treated with or without the ERK inhibitor U0126 and the farnesyl transferase inhibitor tipifarnib. Cells were treated with 10 μm U0126 or 5 μm tipifarnib for 24 h and analyzed for changes in levels of Ras and P-ERK. Our results showed that U0126 indeed inhibits ERK phosphorylation in B103-695 cells (Fig. 8*A*). Studies in nonneuronal systems have shown that treatment with tipifarnib leads to unfarnesylation of Ras, which migrates more slowly on gel than farnesylated active Ras (F-Ras; Alcock et al. 2002; Geryk-Hall et al. 2010). Our analysis showed that tipifarnib treatment led to formation of two Ras-reactive bands, with one showing reduced mobility on gel, indicative of inactivated unfarnesylated Ras (U-Ras; Fig. 8*A*). The cells still showed presence of a farnesylated, fast-migrating band indicative of incomplete inactivation of Ras at the tipifarnib concentration we used. Furthermore, B103-695 cells treated with U0126 and tipifarnib showed a decrease in the levels of cyclin D1 (Fig. 8*A*), demonstrating that this activation occurs downstream of Ras-ERK activation.

Previous studies have shown that Aβ-mediated cytotoxic effects depend on APP expression (Shaked et al. 2006; Sola Vigo et al. 2009); therefore, we tested whether Aβ-mediated Ras-ERK activation is APP dependent and whether this could be attenuated by the inhibitor treatment. Here, we treated both B103 and B103-695 cells with or without 2.5 μm oligomeric Aβ for 24 h and analyzed for changes in P-ERK, total ERK, cyclin D1, and Ras by Western blotting. Our results showed that B103 cells that are null for APP did not show any change in Ras, P-ERK, or cyclin D1 in response to Aβ treatment, unlike B103-695 cells, which showed significant increases in the levels of these proteins (Fig. 8*B–E*). These data therefore demonstrate that APP is necessary for Aβ to elicit activation of Ras-ERK signaling. Furthermore, in the presence of 10 μm U0126, the Aβ-dependent ERK activation and cyclin D1 expression were attenuated (Fig. 8*B–D*), thereby confirming that cyclin D1 activation occurs downstream of ERK activation. Ras levels were also decreased upon treatment with U0126 but not to the extent that was seen with P-ERK or cyclin D1 (Fig. 8*B*, *E*); total levels of ERK were unaffected by the treatment (Fig. 8*B*).

### Cotreatment with U0126 and tipifarnib inhibits Aβ-mediated increase in cyclin D1 and P-APP in primary neurons

**Figure 9.**
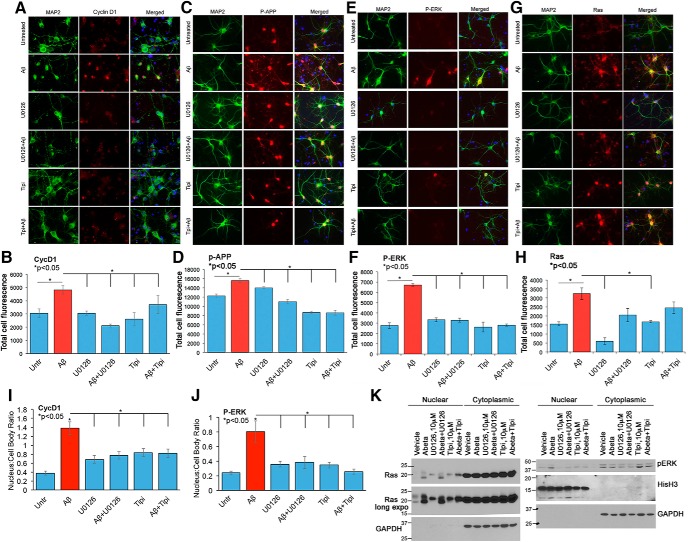
MEK inhibitor U0126 and Ras inhibitor Tipifarnib inhibit Ras-ERK signaling, Cyclin D1 expression, and APP phosphorylation in neurons. ***A–H***, Primary rat neurons were treated with or without 2.5 μm Aβ in the presence or absence of 10 μm U0126 or 5 μm Tipifarnib and analyzed by immunofluorescence using cyclin D1 and MAP2 (***A***), P-APP and MAP2 (***C***), P-ERK and MAP2 (***E***), or Ras and MAP2 (***G***) antibodies. Hoechst was used for visualization of nuclei. The corresponding bar graphs represent the qualitative analysis of the corrected total cell fluorescence for cyclin D1 (***B***), P-APP (***D***), P-ERK (***F***), and Ras (***H***; *n* = 30 neurons/group). ***I***, ***J***, Nuclear versus cytoplasmic ratio of cyclin D1 and P-ERK, respectively, in neurons treated with or without the drugs and Aβ. Statistical analysis was performed using ANOVA, and the data are representative of three independent experiments. Magnification 63×. ***K***, Western blot analysis conducted on nuclear and cytoplasmic isolates from neurons treated with or without 10 μm U0126 and 10 μm Tipifarnib, in the presence or absence of 2.5 μm Aβ show inhibition of Ras farnesylation (left) and P-ERK phosphorylation (right) in the nuclear fraction. Histone H3 antibody was used as nuclear marker and GAPDH as cytoplasmic marker on the reprobed blots.

To determine whether U0126 or tipifarnib interferes with Aβ-dependent changes in Ras, P-ERK, cyclin D1, or P-APP in primary neurons, we treated them with Aβ in the presence or absence of 10 μm U0126 or 5 μm tipifarnib and examined for changes in cellular distribution and levels of the respective proteins using the corresponding antibodies. MAP2 antibody was used as neuronal marker. As shown in Fig. 9*A–D*, the inhibitor treatment attenuated the Aβ-mediated increase in both cyclin D1 and P-APP in the neurons. Although U0126 significantly inhibited P-ERK, the decrease in Ras levels were not significant (Fig. 9*E–H*). Quantitative analysis of the staining in nuclear versus cytoplasmic compartment showed that nuclear levels of P-ERK and cyclin D1 are indeed increased upon Aβ treatment, and this increase is attenuated by the inhibitor treatment (Fig. 9*I*, *J*). Treatment with tipifarnib by itself affected cellular levels of Ras and showed a decrease in the overall cytoplasmic staining, but neurons cotreated with Aβ and tipifarnib did not show a significant decrease in overall staining (Fig. 9*G*, *H*). Tipifarnib treatment seemed to alter the cellular distribution of Ras, which appeared to be nuclear in the treated neurons. To test whether nuclear Ras increased, we conducted Western blot analysis on nuclear and cytoplasmic isolates of neurons treated with or without Aβ in the presence or absence of U0126 and tipifarnib and analyzed for changes in Ras and P-ERK. Two independent experiments were performed in which we used tpifarnib at a concentration of 10 μm. The reason for using 10 μm is that in our B103-695 cells, we noticed that treatment with 5 μm did not completely inhibit the farnesylation of Ras. As shown in Fig. 9*K*, Aβ treatment increased the levels of farnesylated, fast-migrating Ras, indicative of Ras activation. Treatment with 10 μm tipifarnib affected Ras farnesylation (left panel) and showed a slower-migrating band in the nuclear extract from neurons, and samples from neurons cotreated with Aβ and tipifarnib showed inhibition of Ras farnesylation (Fig. 9*K*, left panel). These data imply that the treatment with tipifarnib indeed inhibits Ras activation, whereas total Ras levels may not be affected. Neurons treated with U0126 alone mainly showed unfarnesylated Ras, but upon cotreatment with Aβ, farnesylated Ras levels were increased (Fig. 9*K*, left panel). This is expected, as ERK is a downstream target of Ras and U0126 is a more selective inhibitor of ERK activation by MEK1/2. Cytoplasmic extract from the cells showed very high levels of Ras and did not seem to be affected by the inhibitor treatment (Fig. 9*K*, left panel). Blots reprobed with GAPDH antibody showed presence mainly in the cytoplasmic extracts, although long exposure showed low levels of GAPDH in the nuclear extract as well. Analysis of P-ERK in the same samples (run on a different gel) showed increased nuclear levels in the Aβ-treated samples (Fig. 9*K*, right panel), which was inhibited on cotreatment with both U0126 and tipifarnib. Cytoplasmic P-ERK levels were not increased on Aβ treatment, but U0126 alone and U0126 plus Aβ–treated samples showed a reduction in the levels. Tipifarnib-treated samples showed a slight increase in the cytoplasmic P-ERK levels. Blots reprobed with antibodies to histone H3 and GAPDH showed that these proteins were mainly present in the nuclear and cytoplasmic extracts, respectively, implying that the respective extracts are enriched in mainly nuclear components or cytoplasmic components. Further fractionation of the nuclear components will allow us to determine the nature and localization of active and inactive Ras with nuclear membrane or other nuclear compartments such as nucleolus and nucleoplasm.

**Figure 10.**
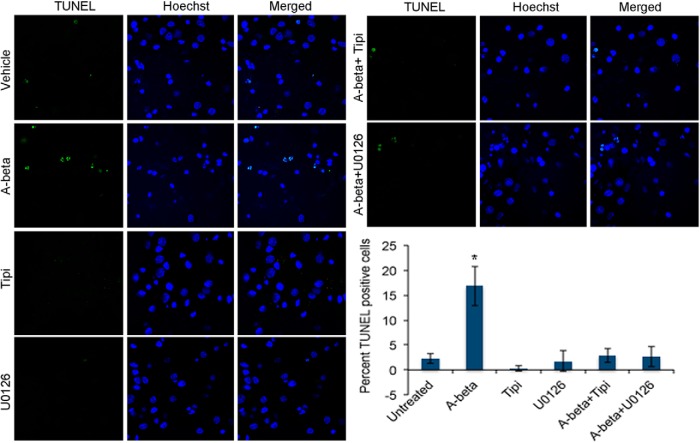
Aβ-mediated apoptosis in neurons is inhibited by U0126 and Tipifarnib treatment. Neurons were treated with or without 2.5 μm Aβ in the presence or absence of 10 μm U0126 or 10 μm Tipifarnib for 24 h and analyzed using TUNEL fluorescence staining kit on a Zeiss fluorescent microscope. Experiment was repeated twice, 100 Hoechst-positive nuclei were counted from three independent fields, and percentage of apoptosis was calculated based on the number of TUNEL-positive nuclei from each field; *p* < 0.05.

To determine whether the inhibitor treatment interferes with Aβ-mediated apoptosis in neurons, we analyzed the neurons using TUNEL apoptosis detection kit. Neurons were incubated with or without 2.5 μm Aβ in the presence or absence of 10 μm U0126 or 10 μm tipifarnib for 24 h, fixed, and stained using the TUNEL staining kit, following the manufacturer’s protocol. Analysis of the neurons showed that these drugs significantly inhibited Aβ-mediated neuronal apoptosis (Fig. 10*A*, *B*). These data imply that interfering with Ras-ERK signaling axis would protect the neurons against Aβ-mediated cytotoxicity.

**Figure 11.**
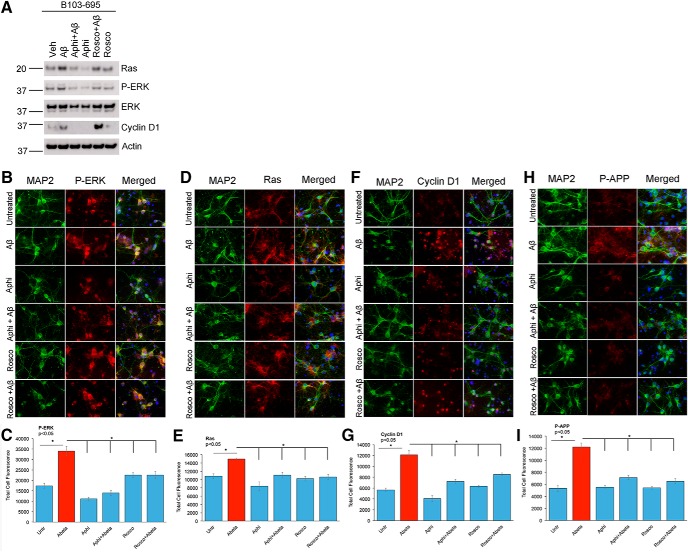
Aphidicolin inhibits Aβ-mediated Ras-MAPK activation, cyclin D1 expression, and APP phosphorylation. ***A***, B103-695 cells were treated with or without 2.5 μm Aβ, in the presence or absence of 5 μg/ml aphidicolin or 20 μm roscovitine for 24 h. On Aβ treatment, as expected expression of Ras and Cyclin D1 as well as phosphorylation of ERK were increased. Aphidicolin treatment diminished both basal and Aβ-induced expression of Ras and Cyclin D1 as well as activation of ERK. Roscovitine did not exhibit any significant effect. Data shown are representative of two independent experiments. ***B–I***, Primary rat neurons were treated with or without 2.5 μm Aβ in the presence or absence of 5 μg/ml aphidicolin or 20 μm roscovitine and analyzed by immunofluorescence using P-ERK and MAP2 (***B***), Ras and MAP2 (***D***), Cyclin D1 and MAP2 (***F***), or P-APP and MAP2 (***H***) antibodies. Hoechst was used for visualization of nuclei. The corresponding bar graphs represent the qualitative analysis of the corrected total cell fluorescence for P-ERK (***C***), Ras (***E***), cyclin D1 (***G***), and P-APP (***I***; *n* = 30 neurons/group). Statistical analysis was performed using ANOVA, and the data are representative of three independent experiments. Magnification 63×.

To test if the Ras-ERK activation can be interrupted by cell cycle inhibitors, we conducted preliminary studies in B103-695 cells treated with 5 μg/mL aphidicolin or 20 μm roscovitine for 24 h in the presence or absence of 2.5 μm Aβ. Aphidicolin is an early S-phase inhibitor that selectively inhibits DNA polymerase alpha, whereas roscovitine is known to inhibit cdk2, cdc2, and cdk5. We observed that aphidicolin treatment decreased the expression of Ras and inhibited ERK phosphorylation (Fig. 11*A*). Furthermore, Aβ-mediated increase in P-ERK and Ras were attenuated upon aphidicolin treatment (Fig. 11*A*). Roscovitine treatment, however, did not show any significant effect on the Aβ-mediated increase in cyclin D1 levels (Fig. 11*A*). These data imply that Ras-ERK activation is an early event and roscovitine-sensitive kinases act downstream of these activation events.

Next we examined whether aphidicolin or roscovitine affects neuronal levels or distribution of Ras, P-ERK, or cyclin D1. Toward this, neurons were treated with or without 2.5 μm Aβ, in the presence or absence of 5 μg/mL aphidicolin or 20 μm roscovitine, for 24 h, fixed and immunostained using cyclin D1, P-APP, Ras, or P-ERK antibodies together with MAP2 antibody as neuronal marker. Similar to our findings in B103-695 cells, aphidicolin treatment significantly decreased Aβ-mediated increase in P-ERK (Fig. 11*B*, *C*), Ras (Fig. 11*D*, *E*), and cyclin D1 (Fig. 11*F*, *G*). The neurons also showed significant decrease in P-APP levels on cotreatment with aphidicolin and Aβ (Fig. 11*H*, *I*). Furthermore, neurons cotreated with Aβ and roscovitine showed significant reduction in the levels of P-APP (Fig. 11*H*, *I*), P-ERK (Fig. 11*B*, *C*), Ras (Fig. 11*D*, *E*), and cyclin D1 (Fig. 11*F*, *G*), but compared with aphidicolin treatment, it was less effective in inhibiting P-ERK and cyclin D1.

Altogether, these results show that APP plays an important role in Aβ-mediated Ras-ERK activation, supporting the hypothesis that APP acts upstream of Ras signaling cascade. We had previously shown that APP is regulated in a mitosis-specific manner and suggested that P-APP association with the cell cycle machinery is indicative of a role for APP in cell cycle deregulation and neurodegeneration in the AD brains (Judge et al. 2011), and the data from the neurons treated with Aβ support this hypothesis.

## Discussion

Genetic evidence for hereditary AD demonstrates genomic duplication of the APP locus and mutations in APP, presenilin 1, and presenilin 2, contributing to altered metabolism of APP and enhanced Aβ formation (Hardy, 1997). Additionally, the ApoE4 allele associated with sporadic AD is linked to increased aggregation and reduced clearance of Aβ (DeMattos et al. 2004; Kim et al. 2009; Wildsmith et al. 2013). It is therefore widely agreed that APP, specifically its toxic Aβ metabolite, is a central player in AD. Soluble, small Aβ oligomers are toxic to neurons (Klein et al. 2001; Walsh and Selkoe, 2004); however, the mechanisms by which oligomeric Aβ leads to synaptic dysfunction and neurodegeneration are not fully understood. We find that both APP and oligomeric Aβ42 modulate the neuronal intracellular signaling, resulting in pathogenic phosphorylation of APP and tau, contributing to neuronal dysfunction and degeneration.

Using SILAC-based proteomic analysis, we previously demonstrated enhanced Ras expression and ERK phosphorylation upon APP expression in B103 rat neuroblastoma cells, suggesting that APP is a modulator of the proliferation-associated Ras-MAPK signaling pathway (Chaput et al. 2012). Others have demonstrated that Ras signaling regulates APP promoter activation and expression (Cosgaya et al. 1996; Mora et al. 2013). Our findings, that APP downregulation leads to reduced Ras expression with a concomitant reduction in ERK phosphorylation, indicate that APP acts as an upstream positive regulator of Ras-dependent signal transduction pathways in neuronal cell lines. We further demonstrate that presence of APP in B103 cells induces not only the expression of Ras but also activation of Ras by demonstrating that it is GTP-bound, which is the active form of Ras. Although this signaling promotes proliferation and transformation in dividing cells, it would likely induce mitotic catastrophe and neurodegeneration in terminally differentiated neurons. Studies on postmortem human brains implicate the Ras-MAPK pathway as an early driver of AD pathology development (Arendt et al. 1995; Gärtner et al. 1995, 1999). Immunocytochemistry has revealed localization of Ras and P-ERK in neurons proximal to plaques and tangles in AD brains, while Western blot analysis has shown increased expression of components of the MAPK signaling cascade in conjunction with increased Aβ levels and phosphorylated tau (Trojanowski et al. 1993; Hyman et al. 1994; Arendt et al. 1995; Gärtner et al. 1995, 1999; McShea et al. 1999; Ferrer et al. 2001). Our results suggest that elevated Ras levels persist from early AD through advanced stages of tau tangle formation, when neurons are most metabolically compromised. Studies in AD brains have shown MAPK activation in response to ATP decline, which contributes to increased tau phosphorylation (Blanchard et al. 1994). Others have demonstrated that P-ERK immunoreactivity in the AD brain follows the path of neuronal degeneration, projecting from trans-entorhinal neurons and progressing with pathology (Pei et al. 2002). Our analysis on control, MCI, and LAD brain samples showed that P-ERK levels varied randomly between groups. This inconsistency could be due to the labile phosphorylation status of ERK in frozen tissue or to possible variations in postmortem tissue collection and processing. Strikingly, increased expression of Ras appears to correlate with Aβ generation and persists through late AD stages, implying a pathologic link between Aβ and altered Ras-MAPK signaling. Additionally, we observed a significant increase in phosphorylated tau in LAD individuals, confirming a correlation and possible molecular link between Aβ, Ras-MAPK signaling, and tau pathology development. Our data show that elevated Aβ levels in the brain correlate to increased expression of Ras and phosphorylation of APP and tau, thus implying a role for proliferative signaling in neurodegeneration observed in AD.

Although the results from brain tissue samples were informative, we were unable to draw a conclusion on the direct role of Aβ in induction of Ras expression or downstream signaling. Aβ treatment can activate the MAPK signaling cascade in acute hippocampal slices as well as primary neurons (Ekinci et al. 1999; Rapoport and Ferreira, 2000; Dineley et al. 2001). ERK activation is a rapid, transient event with peak activation occurring within 2–5 min and returning to baseline after 15 min (Dabrowski et al. 1997; Barge et al. 1998; Porras et al. 1998), and activity exceeding 1 h after exposure to a stimuli is indicative of sustained kinase activation (Dabrowski et al. 1997). Our studies on primary neurons treated with oligomeric Aβ for as little as 5 min to as long as 24 h (only data from 24 h is shown) showed a significant increase in expression of Ras and phosphorylation of ERK only after 6 h of treatment, suggesting that chronic neuronal Aβ exposure elicits sustained MAPK signaling. Additionally, the nuclear translocation of P-ERK confirmed activation of ERK signaling cascade in the neurons in response to Aβ (Jaaro et al. 1997). Enhanced Ras-MAPK signaling axis activates AP-1 and induces expression of cyclin D1 (Balmanno and Cook, 1999), and our studies show that Aβ not only induces Ras-MAPK signaling but also enhances expression and nuclear accumulation of cyclin D1 in neurons, which could be attenuated by inhibition of the Ras-ERK signaling cascade. Because increased nuclear levels of cyclin D1 are associated with cell cycle activation and G1/S progression, we believe a similar mechanism is activated by Aβ in neurons, forcing the neurons to enter cell cycle. However, the lack of functional cell cycle machinery hinders the neurons from undergoing successful division, and alternatively they become vulnerable and undergo neurodegeneration and ultimately death.

We find that Aβ induces neuronal tau hyperphosphorylation, which is consistent with others’ observations (Busciglio et al. 1995; Le et al. 1997; Geula et al. 1998; Takashima et al. 1998; Götz et al. 2001). Although it is likely that P-ERK is contributing to tau phosphorylation (Lu et al. 1993; Raghunandan and Ingram, 1995; Tanaka et al. 1998; Reynolds et al. 2000), tau is a substrate for other kinases such as stress-activated kinase (Utton et al. 1997), cyclin-dependent kinases (Kobayashi et al. 1993), and most notably GSK-3 (Utton et al. 1997). APP is implicated in regulation of GSK-3 activity, and *in vitro* studies have demonstrated that AICD interacts with GSK-3β and induces its activity (Zhou et al. 2012). Similarly, AICD transgenic mice show a dramatic increase in GSK-3 activity (Ryan and Pimplikar, 2005). We found that upon APP knockdown, phosphorylation of GSK-3 is increased, indicative of its reduced activity. Analysis of human brain samples showed a reduction in P-GSK-3 levels in MCI and LAD individuals compared with controls without dementia, suggesting that abnormal GSK-3 activity is a pathogenic event in both early- and late-stage AD. GSK-3 can phosphorylate tau at both primed and unprimed residues (Cho and Johnson, 2004). Thr231 is an important priming residue on tau and is a primary phosphorylation site for GSK-3 (Lin et al. 2007). We found that P-Thr231 levels were significantly elevated in LAD individuals; samples from MCI showed a slight increase that was not significant. PHF-1, however, was detected only in LAD brain samples, which corresponded with high levels of Aβ. Additionally, the phosphorylation of APP at Thr668 was significantly increased in LAD compared to MCI and non-AD individuals, implying a role for activation of Ras-MAPK axis as well as GSK-3 in induction of this phosphorylation.

The role of Aβ in promotion of these signaling events is confirmed by our findings in neurons. Aβ enhanced not only Ras-ERK activation, but also GSK-3 activation, cyclin D1 expression, and nuclear translocation, together with phosphorylation of tau (PHF-1) and APP (Thr668). The finding that Aβ induces APP phosphorylation is novel, and it is likely that aberrant activation of the Ras-MAPK signaling axis and GSK-3 by Aβ-APP signaling is central to the APP and tau phosphorylation, impaired axonal transport, and microtubule destabilization observed in AD.

**Figure 12.**
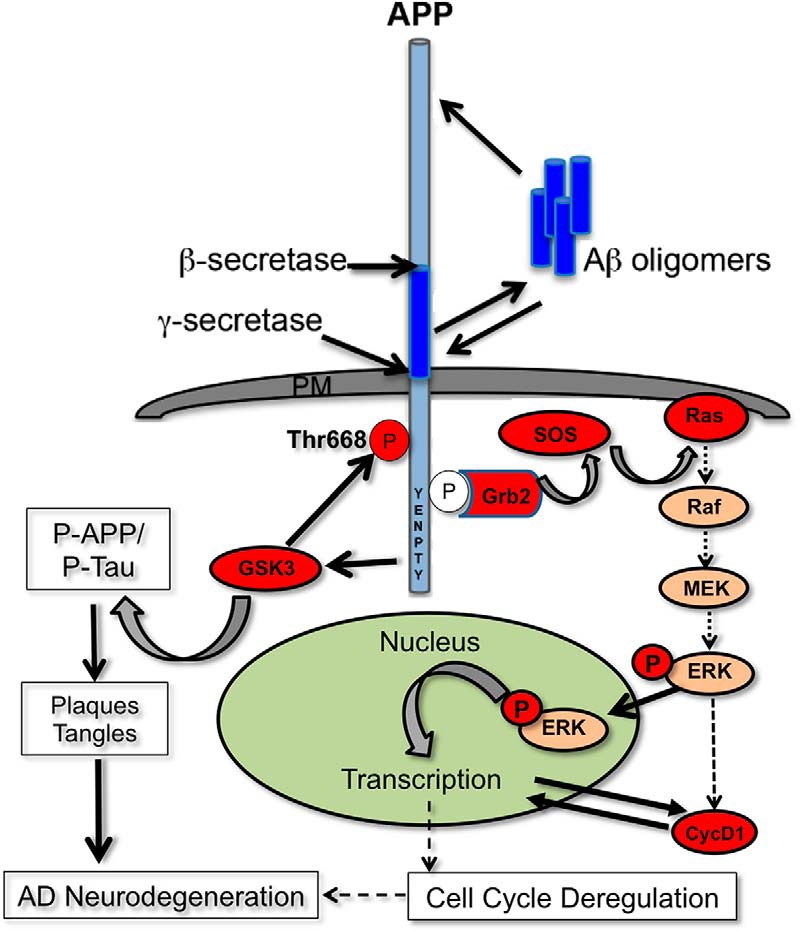
Schematic showing intracellular signaling by APP and Aβ. Sporadic AD and familial AD are associated with increased APP proteolysis and Aβ production or decreased Aβ clearance. Our results show that altered APP expression and Aβ production would enable Aβ-APP signaling and activation of Ras-MAPK signaling axis, which would promote proliferative signaling and neuronal cell cycle entry. Others have shown that growth factor signaling could promote interaction of APP intracellular domain with adaptor proteins, such as Grb2, and recruitment of MAPK signaling components. Additionally, our studies suggest a potential role for APP in GSK3 activation. Activation of ERK and GSK-3 could lead to the pathogenic phosphorylation of both APP and tau. Phosphorylation of APP at Thr668 results in pathogenic processing of APP and enhanced production of Aβ, which feeds back into the vicious cycle, to promote both neuritic plaque and neurofibrillary tangle formation, neurodegeneration, and ultimately neuronal death observed in AD.

In conclusion, our results show that concerted signaling by both APP and Aβ is necessary for aberrant neuronal cell cycle entry. Others have shown that Aβ toxicity is dependent on the presence of APP (Shaked et al. 2006; Sola Vigo et al. 2009), and APP has been implicated as a possible receptor for Aβ (Lorenzo et al. 2000; Kedikian et al. 2010). The cytoplasmic YENPTY motif of APP is known to serve as docking sight for adaptor proteins, Shc and Grb2, which recruits the guanine nucleotide exchange factor SOS2 for activation of Ras-MAPK cascade and intracellular signaling (Nizzari et al. 2007, 2012). Based on our results from APP knockdown studies and inhibitor treatments, we hypothesize that Aβ acts via APP to induce downstream signaling through activation of the Ras-MAPK axis to promote pathologic phosphorylation of both tau and APP (Fig. 12). Phosphorylation of APP not only enhances its proteolysis by BACE to generate more Aβ, but also its association with the centrosomes, implying a role for APP in aberrant cell cycle signaling in the compromised brain regions. Thus, APP plays a central role in promotion of neurodegeneration, through activation of Ras-ERK signaling axis as well as GSK-3, and interfering with these signaling events would impede cell cycle reentry and neurodegeneration observed in AD.

## Synthesis

Reviewing Editor: Lorna Role, Stony Brook Decisions are customarily a result of the Reviewing Editor and the peer reviewers coming together and discussing their recommendations until a consensus is reached. When revisions are invited, a fact-based synthesis statement explaining their decision and outlining what is needed to prepare a revision will be listed below. The following reviewer(s) agreed to reveal their identity: Carol Troy

Overall, although the authors did add the previously requested inhibitor experiments the revised manuscript still does not address the key issue that was raised by the reviewers. Specifically: the data presented are still just correlative. The authors must address this issue as detailed below.

In addition the data provided in the rebuttal letter appear to contradict the text and the pictures shown include multiple panel duplications.

Major issues:

1. Although the inhibitor experiments have now been included, the authors still do not provide any direct measure of neurodegeneration. In fact, there does not appear to be any neuronal degeneration after 24 hr of Aß treatment, and the morphology of the MAP2-stained neurons shown looks quite healthy. Are there fewer neurons after 24 hr of Aß treatment?

a. The authors must provide data (with quantification) that addresses the issue correlation/ causation to establish a link between the Abeta treatments (+/- inhibitors) and a direct measure of neurodegeneration and/or cell death.

b. The authors claim (in direct contrast to the literature) that they do not see any cell death in their primary neuron experiments at 24 hours post Abeta treatment (Figs 4,5, 6, and 9). This discrepancy with the literature must be addressed and the authors must provide data that supports their choice of experimental time points.

I.E.

--- At what point in time post A Beta treatment is cell death/neuronal degeneration observed?

--- Is the cell death / degeneration profile altered by the treatments with the inhibitors? Please quantify.

--- At what time point is the cell death prevented by the inhibitor treatments? This data should be quantified and included.

2. The early time point figure that is provided in the rebuttal letter is presented to show that there is no activation prior to 4-6hr. Actually some of the images appear to show earlier activation, for example both reviewers noted that cyclin D1 and Ras appear increased by 30min. Please address.

Even if the activation doesn't occur until 4-6hr, that is much earlier than the 24hr point used throughout the manuscript. As such : the authors must justify the time points examined.

It is best to study signaling at time points when it is beginning to be able to delineate the hierarchical relationship of the signals to the outcome. Why have the authors used 24 hours as the time point?

The Ras 30 min and 1 hour images appear to be duplicates of one another. Please address this.

The entire pErk and cyclin D panels appear to be duplicated. Please address this.

In addition the reviewers have identified several figure specific issues that must be addressed:

Figure 5, the authors show activation of the ras-Erk pathway and cell cycle activation by looking at nuclear localization of P-Erk and cyclin D1 in primary cortical neurons treated with Aß. While the merged images clearly show increased P-Erk with Aß treatment, the cyclin D1 appears to be already localized to the nucleus in untreated cells, so it's not clear what's being quantified in the graph.

Figure 7, PHF-1 blot appears to show increased levels with Aß treatment, although the graph does not indicate any change, therefore the blot does not appear to be representative.

Figure 9 - In some of the staining and quantification it is not clear what is being quantified. Please clarify.

The total cell fluorescence would appear to indicate the level of protein (does it include all the processes as well as cell body?), but there is also mention of protein distribution, and there is no quantification of nuclear versus cytoplasmic staining. Please provide this quantification.

On page 19 the authors mention that U0126 did not affect Ras distribution, but in figure 9G-H there appears to be a decrease in total Ras staining with Aß+U0126, but no quantification of localization. Please provide quantification of changes in localization.
